# Application of Sparse Linear Discriminant Analysis and Elastic Net for Diagnosis of IgA Nephropathy: Statistical and Biological Viewpoints

**DOI:** 10.29252/.22.6.374

**Published:** 2018-11

**Authors:** Tahereh Mohammadi Majd, Shiva Kalantari, Hadi Raeisi Shahraki, Mohsen Nafar, Afshin Almasi, Shiva Samavat, Mahmoud Parvin, Amirhossein Hashemian

**Affiliations:** 1 Department of Biostatistics and Epidemiology, Kermanshah University of Medical Sciences, School of Public Health, Kermanshah, Iran; 2 Chronic Kidney Disease Research Center, Labbafinejad Hospital, Shahid Beheshti University of Medical Sciences, Tehran, Iran; 3 Department of Biostatistics, School of Medicine, Shiraz University of Medical Sciences, Shiraz, Iran; 4 Urology-Nephrology Research Center, Labbafinejad Hospital, Shahid Beheshti University of Medical Sciences, Tehran, Iran; 5 Department of Biostatistics and Epidemiology, School of Public Health, Kermanshah University of Medical Sciences, Kermanshah, Iran; 6 Department of Nephrology, Labbafinejad Medical Center, Shahid Beheshti University of Medical Sciences, Tehran, Iran; 7 Department of Pathology, Labbafinejad Medical Center, Shahid Beheshti University of Medical Sciences, Tehran, Iran; 8 Research Center for Environmental Determinants of Health (RCEDH), Kermanshah University of Medical Sciences, Kermanshah, Iran; 9 Department of Biostatistics and Epidemiology, Faculty of Health, Kermanshah University of Medical Sciences, Kermanshah, Iran

**Keywords:** Biomarker, Diagnosis, IgA nephropathy, Proteomics

## Abstract

**Background:**

IgA nephropathy (IgAN) is the most common primary glomerulonephritis diagnosed based on renal biopsy. Mesangial IgA deposits along with the proliferation of mesangial cells are the histologic hallmark of IgAN. Non-invasive diagnostic tools may help to prompt diagnosis and therapy. The discovery of potential and reliable urinary biomarkers for diagnosis of IgAN depends on applying robust and suitable models. Applying two multivariate modeling methods on a urine proteomic dataset were obtained from IgAN patients, and comparison of the results of these methods were the purpose of this study.

**Methods:**

Two models were constructed for urinary protein profiles of 13 patients and 8 healthy individuals, based on sparse linear discriminant analysis (SLDA) and elastic net (EN) regression methods. A panel of selected biomarkers with the best coefficients were proposed and further analyzed for biological relevance using functional annotation and pathway analysis.

**Results:**

Transferrin, α1-antitrypsin, and albumin fragments were the most important up-regulated biomarkers, while fibulin-5, YIP1 family member 3, prasoposin, and osteopontin were the most important down-regulated biomarkers. Pathway analysis revealed that complement and coagulation cascades and extracellular matrix-receptor interaction pathways impaired in the pathogenesis of IgAN.

**Conclusion:**

SLDA and EN had an equal importance for diagnosis of IgAN and were useful methods for exploring and processing proteomic data. In addition, the suggested biomarkers are reliable candidates for further validation to non-invasive diagnose of IgAN based on urine examination.

## INTRODUCTION

IgA nephropathy (IgAN) is the most common primary glomerulonephritis in the world[1]. IgAN is diagnosed based on invasive renal biopsy by evidence of mesangial IgA deposits along with proliferation of mesangial cells[1,2]. The disease has a wide spectrum of clinical presentations, ranging from asymptomatic microscopic hematuria in mild stages to macroscopic hematuria with heavy proteinuria in a more severe course that results in rapid deterioration of renal function[3]. Approximately 20-40% of patients with IgAN end up to end-stage renal disease within 20 years after diagnosis[4]. Thus, a timely and non-invasive diagnosis and a more clear insight of pathogenesis may help to save patients from kidney failure. Recently, numerous high-throughput studies have attempted to identify more applicable, reliable and non-invasive biomarkers for renal diseases[5-10], instead of the application of non-specific biochemical factors and invasive diagnostic methods. Several omics analyses have been used for identifying biomarkers for IgAN[6,11-13]; however, the reliable non-invasive diagnostic biomarkers and the impaired biological pathways involved in the process of the disease need to be determined. These high-throughput data, which are obtained via omics technologies (e.g. proteomics, transcriptomics, metabolomics, genomics, meta-genomics, epigenomics, etc.), are characterized by the dimensionality and complexity[14]. Dimensionality refers to hundreds to thousands of variables or “p” (e.g. genes, proteins, metabolites, etc.), whereas the number of samples or “n” are relatively small[15]. When these data are high-dimensional and multicollineared, the univariate analysis, which ignores the covariance between the variables and is prone to false positives, seems not to be appropriate[16]. Therefore, univariate analysis could not reflect the biological relationship between the correlated variables.

To address the extraction of biological information due to joint impacts of variables and dimension reduction, different feature selection and classification methods such as least absolute shrinkage and selection operator (LASSO)[17], adaptive LASSO[18], partial least squares discriminant analysis (PLS-DA)[9], sparse linear discriminant analysis (SLDA)[19], elastic net-type regularized regression[20], and smoothly clipped absolute deviation (SCAD)[21] have been established over the past decades. The judge on the applicability of the analysis method depends on the aim of study and quality of data. Elastic net and SLDA are two of the popular methods for high-throughput data analysis. Elastic net is a linear combination of Ridge[22] and Lasso[17] with both shrinkage and automatic biomarker selection, whereas SLDA performs discriminant analysis with penalty coefficients to obtain sparsity according to chosen variables[17,20]. We selected these two methods because elastic net has advantages of both Ridge and LASSO regression (when the penalty weight (α) is equal to 1, it produces the LASSO regression, and when α = 0, it produces Ridge regression. Accordingly, in elastic net, penalty weight is 0 ≤ α ≤ 1. Furthermore, the results of analyzing high-throughput data are more interpretable than using ordinary linear discriminant analysis (LDA), and it is extensible to non-normal data. In this study, we compared the efficiency of these two methodologies to obtain reliable diagnostic biomarkers for IgAN. The results were then compared with PLS-DA in our previous paper[10]; a panel of selected biomarkers based on these three models are suggested, and biological relevance of identified biomarkers are discussed.

## MATERIALS AND METHODS

### Description of urine proteome dataset

In order to compare the performance of two methods (SLDA and elastic net) for diagnosis of IgAN, urine protein profile of patients with IgAN and of healthy subjects, which was obtained using nanoscale liquid chromatography-high resolution tandem mass spectrometry, was used[10]. The dataset is made up of the urinary protein profiles of 13 patients (11 men and 2 women) and 8 (6 men and 2 women) healthy volunteers as the control group with the mean age of 33 and 34.5 years of age, respectively, each with 493 variables. The samples were collected from patients who were referred to Labbafinejad Medical Center in Tehran (Iran) during 2011 to 2012. Demographic, histopathologic and clinical data of the patients are tabulated in Table 1.

**Table 1:** Demographic, histopathologic, and laboratory data of patients

No.	Age (Y)	Sex	MEST score	WHO Stage	MH	EnH (%)	ExH (%)	IFTA (%)	Sclerosis (%)	Inflammation (%)	sCr (mg/dl)	Proteinuria (mg/24 h)
1	28	M	M1E0S1T2	V	severe	NO	NO	80	83	25	4.6	2330
2	34	M	M1 S1 E0 T1	V	severe	NO	NO	60	73	25	2.3	2640
3	18	M	M1 S0 E0 T1	III	mild	NO	NO	20	NO	50	0.9	1000
4	45	M	M1 S1 E1 T0	III	mild	<50	NO	15	29	NO	1.3	720
5	47	M	M1 S0 E0 T0	II	mild	NO	NO	10	NO	25	1.1	6420
6	41	M	M1 S0 E0 T0	II-III	moderate	NO	NO	NO	5	NO	0.5	520
7	29	M	M1 S1 E1 T2	V	mild	<50	<50	80	50	>50	7.7	7020
8	28	F	M0 S1 E0 T0	III	mild	NO	NO	20	NO	NO	0.7	1680
9	34	M	M1 S0 E0 T0	II-III	moderate	NO	NO	5	14	NO	1	1310
10	32	M	M1 S1 E0 T1	IV	moderate	NO	<50	40	30	35	1.8	4100
11	29	M	M1 S0 E1 T2	V	severe	NO	NO	80	78	<25	5	6000
12	23	F	M1 S1 E1 T1	III	moderate	<50	NO	20	15	NO	1.2	800
13	51	M	M1 S1 E0 T0	II	moderate	NO	NO	15	NO	NO	1.7	4600

No. number of patients; MH, mesangial hypercellularity; EnH, endo-capillary hypercellularity; ExH, extra-capillary hypercellularity; IFTA, interstitial fibrosis-tubular atrophy; sCr, serum creatinine. MEST score is acronym of mesangial proliferation, endocapillary hypercellularity, sclerosis/adhesions, tubular atrophy/interstitial fibrosis.

### Sparse linear discriminant analysis (SLDA)

LDA, which uses a linear combination of features as the criterion for classification, has often been shown to produce the best classification results for low dimensional data[23-26]. Although LDA is popular because of its simplicity and predictive ability, it fails to work when there are too many correlated predictors, and when the number of features is exceeded than sample size[19,26,27]. In this condition, which is mainly true in omics data, the result is difficult to interpret. To solve the limitations of LDA, the SLDA was defined. SLDA led to have a model with more accurate estimation and more accurate prediction capabilities as well as a higher power. Suppose that X is an n × p matrix, where p is the number of predictor variables, and n is the number of samples. Also, x_i_ and x_j_ are i^th^ row and j^th^ column of the matrix X, respectively. The number of observations in k^th^ group is shown with C_k_, that is a subset of[12] 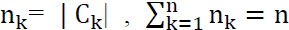. An estimate for the within-class covariance matrix 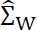 and an estimate for the covariance matrix between classes 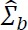 can be defined as follows, respectively:


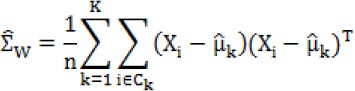



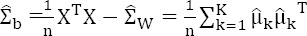


That 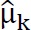 is the sample mean vector for k^th^ class. Finally, the following command acquires k^th^ penalized discriminant vector:


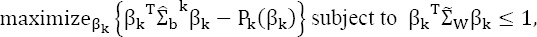


Where P_k_ is a convex penalty function on the k^th^ discriminant vector, and 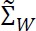 is a positive definite estimate for Σ_W_. SLDA analysis was performed using PenalizedLDA package.

### Elastic net regression analysis

The elastic net-type regularized regression (e.g., ridge[22], lasso[17], elastic net[20], etc.) is a popular data analysis method for identifying features based on high, dimensional omics dataset[28]. For less variability and construction a reliable model in case of datasets with correlated features, elastic net regression reduce the variance of the model by constraining the size of the regression coefficients[29]. This method of feature selection is called regularization based on imposed constraint (i.e. penalty) on the coefficients. The elastic net regression analysis acts as a bridge between the ridge and lasso regressions. The L_2_-norm (the sum of squared values), and the L_1_-norm (the sum of absolute values) of the coefficients are considered as penalty in ridge and lasso regressions, respectively, while a hybrid of these two penalties is considered in elastic net regression.


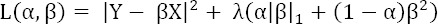


Where |β|_1_ is L_1_-norm of the vector of regression coefficients, and |Y−βX|^2^ is the sum of the squared residuals from the fit[29]. The value of the λ and α parameters can be estimated by performing cross-validation method. For n observation, if β^T^=(β_1_,β_2_,...,β_p_) is a vector of p variables, elastic net logistic regression is defined as follow:


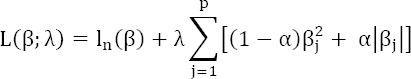


Where ln (β) is a maximum likelihood estimator for logistic regression, α is a value between zero and one, and λ is a positive constant called tuning parameter that manages the shrinkage degree[29]. Elastic net regression was performed using package glmnet in R 3.3.1 software. Parameters for evaluating the agreement of the models, such as cure agreement and kappa, were calculated, and Bland-Altman plot was drown. The area under curve (AUC) values was calculated using receiver operating characteristic (ROC) curves for evaluation the accuracy of the models. In order to estimate the optimum amounts of shrinkage in both elastic net and SLDA methods, fivefold cross validation was used. In addition, standard error of coefficients was obtained using 500 times replication of bootstrap methods. Bland-Altman plot was drawn in MedCalc 14.0 software, and the other calculations were performed using R 3.3.1 software.

### Functional analysis of identified biomarkers

To further understand the biological relevance of the characterized biomarkers, we performed gene ontology analysis using Cytoscape v 3.4.0 software and a Cytoscape plug-in named ClueGO (version 2.2.5)[30,31]. ClueGO is widely used for analysis and visualization of functionally related genes. Gene ontology analysis composed of three terms, including “biological process”, “cellular component”, and “molecular function” was performed, and the enriched pathways in Kyoto Encyclopedia of Genes and Genomes database (http://www.genome.jp/kegg/) were also identified. The statistical test used for the enrichment was based on a two-sided hypergeometric option with a Benjamini-Hochberg correction, a *p* value less than 0.05, and a kappa score threshold of 0.4. The minimum number of genes was considered 3.

## RESULTS

### Biomarker identification based on elastic net and SLDA models

**Fig. 1:**
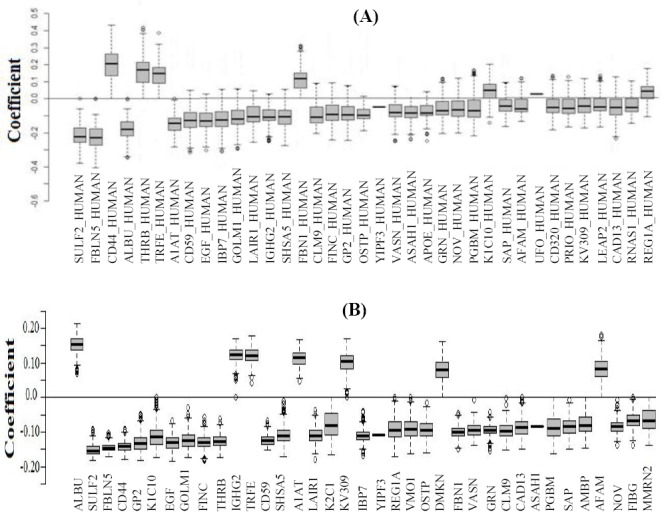
The coefficients of (A) elastic net and (B) SLDA extracted in 500 times bootstrap method.

In this study, we examined the effect of 493 variables in urinary protein profile of IgAN patients and healthy subjects. Univariate analysis using Mann-Whitney test revealed that there was a significant difference (*p* < 0.05) between the case and control groups in 144 out of 493 variables (the results not shown). Because the sample size was small, we directly used fivefold cross-validation to determine the training data and the test data and selected the best parameters (e.g. λ and α) for the methods. For assessing simultaneous effects of aforementioned variables on IgAN disease, elastic net and SLDA models were fitted based on λ = 0.005 and λ = 0.06, respectively. The results of two models indicated that 133 out of 493 variables were effective in discrimination of IgAN in SLDA model, whereas 120 predictive variables were important in elastic net model. Summary of models are shown in Table 2. In this Table, 36 and 37 most important variables in terms of the highest coefficient were reported as discriminative diagnostic biomarkers between two groups for elastic net and SLDA models, respectively. The coefficients of elastic net regression and SLDA for the most effective variables in bootstrap method are shown in Figure 1. There was a good agreement between two models since 30 of selected biomarkers were identical (Table 3), and cure agreement and kappa were 90% and 75%, respectively.

**Table 2:** Summary of models

Model	Zero	Non-zero	Total
SLDA	360	133	493
EN	373	120	493

**Table 3:** List of identical biomarkers that were significant in both models

Protein ID	SLDA		Elastic net		FC	Direction
	
	Coefficient	SE	Coefficient	SE		
TRFE	0.21	0.16	0.14	0.02	7.8	↑
A1AT	0.19	0.14	0.13	0.02	7.7	↑
ALBU	0.24	0.20	0.19	0.02	4.3	↑
AFAM	0.08	0.07	0.10	0.03	3.7	↑
IGHG2	0.14	0.10	0.15	0.02	2.9	↑
KV309	0.08	0.06	0.13	0.03	2.6	↑
FBLN5	-0.26	0.19	-0.17	0.01	11.8	↓
YIPF3	-0.13	0.10	-0.12	0.02	10.6	↓
SAP	-0.08	0.07	-0.10	0.03	10.3	↓
OSTP	-0.13	0.09	-0.11	0.02	9.4	↓
SULF2	-0.30	0.28	-0.18	0.02	9.0	↓
CD44	-0.26	0.21	-0.17	0.02	8.9	↓
CD59	-0.17	0.14	-0.14	0.02	7.5	↓
K1C10	-0.09	0.08	-0.16	0.03	4.7	↓
FBN1	-0.14	0.11	-0.11	0.02	4.2	↓
IBP7	-0.16	0.11	-0.13	0.02	4.2	↓
VASN	-0.12	0.09	-0.11	0.02	3.6	↓
LAIR1	-0.16	0.11	-0.13	0.02	3.6	↓
GP2	-0.13	0.11	-0.17	0.02	3.3	↓
REG1A	-0.08	0.06	-0.12	0.03	3.1	↓
SHSA5	-0.14	0.10	-0.13	0.02	3.1	↓
CLM9	-0.14	0.11	-0.11	0.02	2.7	↓
FINC	-0.14	0.10	-0.16	0.02	2.6	↓
PGBM	-0.09	0.07	-0.11	0.03	2.5	↓
GRN	-0.10	0.08	-0.11	0.02	2.4	↓
CAD13	-0.08	0.06	-0.11	0.03	2.2	↓
EGF	-0.17	0.13	-0.16	0.02	2.2	↓
ASAH1	-0.11	0.09	-0.11	0.02	2.1	↓
GOLM1	-0.16	0.12	-0.16	0.02	1.9	↓
NOV	-0.10	0.06	-0.10	0.02	1.9	↓

SE, standard error; FC, fold change; ↑, up-regulation; ↓, down-regulation

**Fig. 2:**
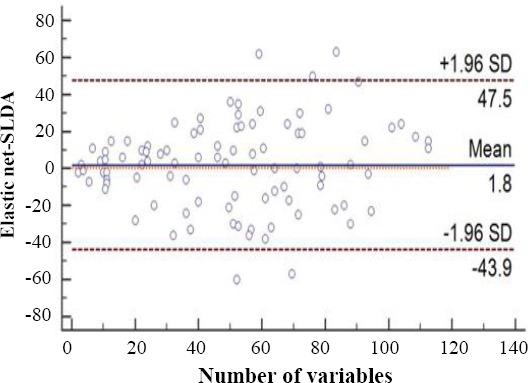
Bland-Altman plot for SLDA and elastic net models.

Bland-Altman plot was drawn based on the rank of the importance variables for emphasizing the agreement between the models (Fig. 2). Table 4 describes the variables that were different between the models. ROC curve revealed that the AUC for both models were 100%, and no misclassification was observed. Also, the optimum cut-off point for probability of IgAN was obtained as 0.52 in elastic net regression using ROC curve analysis. We considered the protein IDs from the Table 2 as the most important diagnostic biomarkers, because these biomarkers were remained significant with high coefficient value in both models. The top four up- and down-regulated biomarkers are tabulated in Table 5 with calculated sensitivity and specificity.

**Table 4:** List of significant biomarkers that were different between two models

SLDA					Elastic net				
	
Protein ID	Coefficient	SE	FC	Direction	Protein ID	Coefficient	SE	FC	Direction
THRB	-0.22	0.16	4.2	↓	K2C1	-0.13	0.04	1.1	↓
APOE	-0.11	0.08	2.5	↓	VMO1	-0.12	0.03	3.8	↓
UFO	-0.08	0.05	4.7	↓	DMKN	0.11	0.03	1.9	↑
CD320	-0.08	0.06	2.0	↓	AMBP	-0.10	0.03	2.5	↓
PRIO	-0.08	0.07	2.4	↓	FIBG	-0.10	0.02	1.9	↓
LEAP2	-0.08	0.07	4.1	↓	MMRN2	-0.09	0.03	1.9	↓
RNAS1	-0.08	0.10	3.0	↓					

SE, standard error; FC, fold change; ↑, up-regulation; ↓, down-regulation

**Table 5:** Panel of suggested diagnostic biomarkers for IgA nephropathy based on elastic net, SLDA, and comparison with PLS-DA

Biomarker	Sensitivity (%)	Specificity (%)
TRFE	100	100
A1AT	100	100
ALBU	100	100
AFAM	100	92.31
FBLN5	100	100
YIPF3	100	100
SAP	75	100
OSTP	100	92.31
GP2	87.5	100
CLM9	100	92.31
VASN	87.5	100
CD44	100	100
EGF	100	100
FBN1	100	100

### Functional annotation of IgAN-related biomarkers

**Fig. 3:**
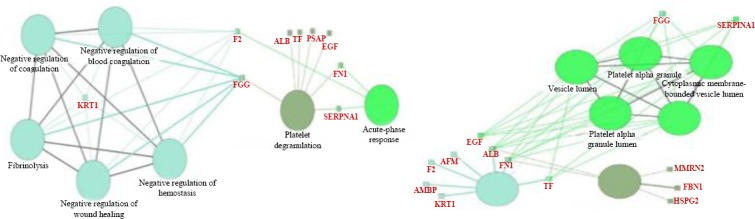
The proteins encompassed by enriched biological processes (A) and cellular component (B), using Cytoscape v 3.4.0 software. The large circles represent biological processes (A) and cellular component (B), and the small rectangles represent the proteins. The circles with the same colors have the same level of significance, and therefore they are in the same GO group. In A, the blue, green, and gray circles show *p* = 35.0 × 10^-6^, *p* = 24×10^-6^, *p* = 3.1 × 10^-9^, respectively. In B, the green circles represent *p* = 15 × 10^-9.^ The blue circle represents *p* value = 250 × 10^-12^, and the grey circle represents *p* value = 2.1 × 10^-6^.

**Fig. 4:**
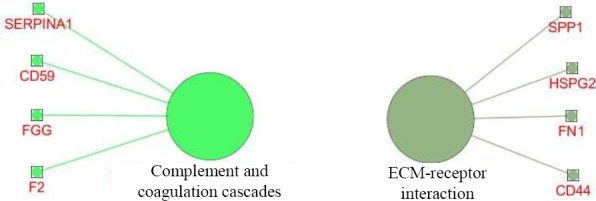
Enriched pathways involved in pathogenesis of IgAN. The large circles represent pathways, and the small rectangles represent the proteins.

To better understand the biological functions of the most important discriminative proteins, we carried out functional enrichment analyses via ClueGO. The integrative list of the biomarkers identified by two models composed of 43 proteins (Tables 2 and 3) was analyzed by GO terms and pathways. Only GO terms with a corrected *p* value < 0.05 were considered statistically significant. Three major groups, including acute-phase response (*p* = 24 × 10^-6^), fibrinolysis (*p* = 35.0 × 10^-6^), and platelet degranulation (*p* = 3.1 × 10^-9^), encompassing seven terms of biological process were remained significant. The significant terms and their nodes are displayed in Figure 3A. As shown in Figure 3B, basement membrane (*p* = 2.1 × 10^-6^), secretory granule lumen (*p* = 15 × 10^-9^), and blood microparticle (*p* = 250 × 10^-12^) were the important biomarkers enriched in three clusters composed of seven terms of cellular component. The GO levels were different for each term, and vary between 2 to 12. However, each term was reported under multiple levels from general nodes (higher parents) to more specific child nodes (lower nodes). In contrast, no GO term was enriched for the categories of molecular function. The results of pathway enrichment analysis revealed two significant pathways: complement and coagulation cascades (*p* = 1.9 × 10^-5^) and extracellular matrix (ECM)-receptor interaction (*p* = 1.9 ×10^-5^). The enriched pathways and their nodes are displayed in Figure 4.

## DISCUSSION

IgAN is the most common type of primary glomerulonephritis worldwide. This disease has a significant morbidity and leads to end-stage renal disease in about 40% of patients within 20 years of diagnosis[32]. The histopathologic hallmark of IgAN is the dominant or co-dominant deposition of IgA in the glomerular mesangium that is usually accompanied by mesangial cellular proliferation and expansion of the ECM[33]. Although renal biopsy involves a risk of morbidity due to bleeding complications, it has currently been considered the only reliable diagnostic approach for IgAN as other glomerular diseases[12]. Nonetheless, other alternative non-invasive approaches appear to be necessary for reducing the difficulties of biopsy and improving its reliability. The ideal non-invasive method is analyzing urine specimen using omics technologies such as urinary proteomics and metabolomics. However, analyzing high-dimensional data producing by these techniques are challenging and require appropriate multivariate modeling. Two popular multivariate models that are compatible with the nature of high-dimensional data are SLDA and elastic net. We have compared the application of these two robust models to identify diagnostic protein biomarkers for IgAN. The good reproducibility of the results from two models indicates the efficiency and the power of penalty-based regression models for biomarker discovery researches. Since the accuracy of both models was 100%, there was no superiority between them in this case. Therefore, we highlighted the importance of a subset of 30 shared biomarkers obtained using two models with highest regression coefficients and less error (Table 3). The top four overrepresented and underrepresented biomarkers in this list were as follows, respectively: transferrin (TRFE), α1-antitrypsin (A1AT), albumin (ALBU), afamin (AFAM), and fibulin-5 (FBLN5), YIP1 family member 3 (YIPF3), prasoposin (SAP), and osteopontin (OSTP).

The urinary excretion of the overexpressed biomarkers in patients with IgAN have been reported earlier[9,34-36], and our results validate the role of these molecules as biomarkers. However, these are not specific for IgAN and have been detected in other glomerular diseases[5,37]. The amount of excretion of proteins in IgAN may be different from other glomerular disease. Further studies requires for proving this hypothesis. Among the overrepresented biomarkers, the evidence on increased urinary A1AT in IgAN is more reasonable. A1AT, also known as SERPINA1, is a serine protease inhibitor that is mainly produced by liver cells, but it is also synthesized by macrophages, neutrophils, activated lymphocytes, and intestinal epithelial cells[9]. Injured renal tubular epithelial cells also can synthesize A1AT in response to tubulointerstitial damage[38]; hence, A1AT is absence in normal urine but detectable in other renal diseases[39]. The possible relevant reasons for A1AT up-regulation in IgAN are: (I) enhancement the synthesis of TRFE receptor or CD71, that is like a receptor for aberrant IgA on the mesangial cells in the kidney tissue of IgAN patients[40], (II) inhibition of thrombin (coagulation factor II), and induction of fibrinolysis during blood coagulation by A1AT in response to hematuria, which is typically presents in IgAN[31,41], and (III) inactivation of proteinases secreted from inflammatory cells, such as neutrophils, by A1AT in IgAN patients[42]. In addition, our finding is in agreement with Neprasova *et al*.[11] and Prikryl *et al*.[42] resultsthat had detected A1AT in the urine of patients with IgAN using labeling proteomic techniques.

There is less evidence on the biomarker role of proteins that suggested as down-regulated candidates for IgAN unless OSTP, and they are presented in this paper for the first time. FBLN5 that is named as FBLN5 is associated with elastic fiber formation as a Ca^2^-dependent elastin-binding protein and presents in interstitial renal tissue, as well as distal renal tubules[43,44]. It has a role in tissue repair, the inhibition of endogenous angiogenesis, and remodeling in response to oxidative stress-mediated renal damage[43,44]. Down-regulation of FBLN5 indicates the low capacity of repair of the injuries mediated by oxidative stress in IgAN.

SAP is a highly conserved lysosomal glycoprotein hydrolases involved in the hydrolysis of sphingo-lipids[45]. Accordingly, it can be postulated that the hyperlipidemia associated with IgAN with nephrotic range proteinuria might be occurred because of the decreased level of this protein in renal tissue of IgAN patients. In addition, the impaired lysosomal pathway that was reported in IgAN based on urine proteomic data[36] rationalizes the contribution of SAP as a lysosomal enzyme in the pathogenesis of IgAN.

Significant reduction of urinary excretion of OSTP in our analysis corresponds with a previous study[46], which highlight its potential role as a IgAN biomarker. A possible explanation for underrepresentation of OSTP in IgAN could be the cleavage of this protein by serine proteases that their activities increase in IgAN[47]. Furthermore, there is a strong correlation between the excretion of OSTP and its receptor CD44 (OSTP receptor) in our dataset (r = 0.93) that has a known contribution in IgAN[48]. CD44 is one of the seven biomarkers (i.e. IGHG2, CD44, VASN, GP2, SHSA5, CLM9, and EGF) that were remained significant in three-type analysis in the present and our previous paper[10], using SLDA, elastic net, and PLS-DA. These sets of biomarkers have been validated statistically and are important targets for experimental validation in the future studies. The contribution of and changes in the urinary level of YIPF3, a natural killer specific antigen, in patients with IgAN is reported here for the first time, and its role in pathogenesis of IgAN needs complementary experiments.

**Fig. 5:**
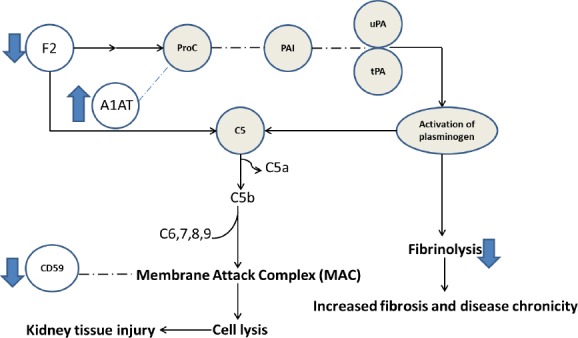
The hypothetical mechanism of renal damage in IgAN mediated by coagulation and complement pathway. The open circles with their direction of changes that are shown by arrows represent the significant proteins that were detected in our dataset. The solid circles represent the mediators that were not in our dataset; nevertheless, connection of the detected biomarkers (in the open circles) to impaired pathways resulted in kidney injury based on literature. ProC, activated protein C; PAI, plasminogen activator inhibitor; 1AT, α1-antitrypsin; uPA, urokinase-type *plasminogen* activator; tPA, tissue-type plasminogen activator; F2, prothrombin. C5a, complement factor 5a; C5b, complement factor 5b; C6,7,8, and 9, complement factors 6,7,8, and 9, respectively.

Investigation of the gene ontology and pathways that enriched in the list of significant biomarkers paved the way to translate invisible information into interpretable biological information. Involvement of acute phase response and processes related to coagulation in IgAN that have been enriched in the dataset is similar to previous studies[49-51]. The role of coagulation pathway in pathogenesis of renal diseases, its crosstalk with complement pathway, and its downstream effects on glycocalyx and formation of ECM are noteworthy nowadays[52]. Therefore, the enrichment of “complement and coagulation cascades” and ECM-receptor interaction pathways is highly relevant to the pathogenic process of IgAN. The important encompassing nodes of these pathways have been described in Figure 4. The possible mechanism of fibrosis and renal damage in IgAN based on our findings might be as follows: down-regulation of F2 in association of up-regulation of A1AT leads to a decrease in the production of activated protein C, which results in the reduction of fibrinolysis. Decrease in fibrinolysis process leads to fibrosis and chronicity. Hence, the inhibitory effect of aPC is removed from plasminogen activator inhibitor (PAI), which exerts the inhibitory effect of PAI on plasminogen activator and downstream production of plasmin. Thus, fibrinolysis process mediated by plasmin decreases, and accumulation of fibrin leads to renal damage and chronicity. Decreased level of fibrinogen gamma chain, as a product of fibrinolysis in our dataset (Table 4), supports this hypothesis. The definition of members of coagulation pathway mentioned above has been reviewed by Madhusudhan *et al*[50]. The hypothetical mechanism of renal damage by coagulation and complement pathway is summarized in Figure 5. Furthermore, down-regulation of CD59, an inhibitor of membrane attack complex in complement pathway[53], may result in exacerbation of renal injury.

Decreased excretion of several involved molecules in ECM-receptor interaction pathway in comparison with normal subjects indicates the instability of glycocalyx in the disease process. Glycocalyx injury will affect a broad spectrum of endothelial function including filtration barrier that leads to proteinuria[54]. In addition, a correlation between endothelial cell injury and renal dysfunction in patients with IgAN has been reported[54]. Therefore, it could be suggested that endothelial injury in IgAN may result in the degradation of glycocalyx in kidney tissue, which is recognized by significant changes (down-regulation) of several elements of ECM that contributes to glycocalyx such as CD44, FN1 (fibronectin), SPP1 (OSTP), and HSPG2 (perlecan), as shown in Figure 4.

In conclusion, functional analysis of the important biomarkers from SLDA and elastic net models revealed that biological processes related to coagulation and acute phase response are involved in the pathogenesis of IgAN. Furthermore, complement and coagulation cascade and ECM-receptor interaction are the two important pathways that impaired in IgAN.
